# An Experimental Design Approach to Quantitative Expression for Quality Control of a Multicomponent Antidiabetic Formulation by the HILIC Method

**DOI:** 10.3390/molecules27103135

**Published:** 2022-05-13

**Authors:** Mahesh Attimarad, Katharigatta Narayanaswamy Venugopala, Muhammad S. Chohan, Marysheela David, Efren II Plaza Molina, Nagaraja Sreeharsha, Anroop Balachandran Nair, Christophe Tratrat, Abdulrahman Ibrahim Altaysan, Abdulmalek Ahmed Balgoname

**Affiliations:** 1 Department of Pharmaceutical Sciences, College of Clinical Pharmacy, King Faisal University, Al-Ahsa 31982, Saudi Arabia; kvenugopala@kfu.edu.sa (K.N.V.); sharsha@kfu.edu.sa (N.S.); anair@kfu.edu.sa (A.B.N.); c_tratrat@kfu.edu.sa (C.T.); aaltaysan@kfu.edu.sa (A.I.A.); a.balgoname@gmail.com (A.A.B.); 2 Department of Biotechnology and Food Technology, Faculty of Applied Sciences, Durban University of Technology, Durban 4000, South Africa; 3 Department of Biomedical Sciences, College of Clinical Pharmacy, King Faisal University, Al-Ahsa 31982, Saudi Arabia; mshwhan@kfu.edu.sa; 4 Department of Nursing, College of Applied Medical Sciences, King Faisal University, Al-Ahsa 31982, Saudi Arabia; mdavid@kfu.edu.sa; 5 Department of Pharmacy Practice, College of Clinical Pharmacy, King Faisal University, Al-Ahsa 31982, Saudi Arabia; emolina@kfu.edu.sa; 6 Department of Pharmaceutics, Vidya Siri College of Pharmacy, Off Sarjapura Road, Bangalore 560035, India

**Keywords:** quality by design, HILIC, chromatography, remogliflozin, vildagliptin, metformin, validation, optimization

## Abstract

A rapid and reproducible hydrophilic liquid chromatography (HILIC) process was established for concomitant determination of remogliflozin etabonate (RE), vildagliptin (VD), and metformin (MF) in a formulation. A face-centered central composite experimental design was employed to optimize and predict the chromatographic condition by statistically studying the surface response model and design space with desirability close to one. A HILIC column with a simple mobile phase of acetonitrile (65% *v*/*v*) and 20 mM phosphate buffer (35% *v*/*v*, pH 6, controlled with orthophosphoric acid) was used to separate RE, VD, and MF. RE, VD, and MF were separated in 3.6 min using an isocratic mode mobile phase flow at a flow rate of 1.4 mL at room temperature, and the analytes were examined by recording the absorption at 210 nm. The developed HILIC method was thoroughly validated for all parameters recommended by ICH, and linearity was observed in the ranges 20–150 µg/mL, 10–75 µg/mL, and 50–750 µg/mL for RE, VD, and MF, respectively, along with excellent regression coefficients (*r*^2^ > 0.999). The calculated percentage relative deviation and relative error ascertained the precision and accuracy of the method. The selectivity and accuracy were further confirmed by the high percentage recovery of added standard drugs to the formulation using the standard addition technique. The robustness of the HILIC processes was confirmed by developing a half-normal probability plot and Pareto chart, as the slight variation of a single factor had no significant influence on the assay outcomes. Utilization of the optimized HILIC procedure for concurrent quantification of RE, VD, and MF in solid dosage forms showed accurate and reproducible results. Hence, the fast HILIC method can be regularly employed for the quality assurance of pharmaceutical preparations comprising RE, VD, and MF.

## 1. Introduction

**Figure 1:**
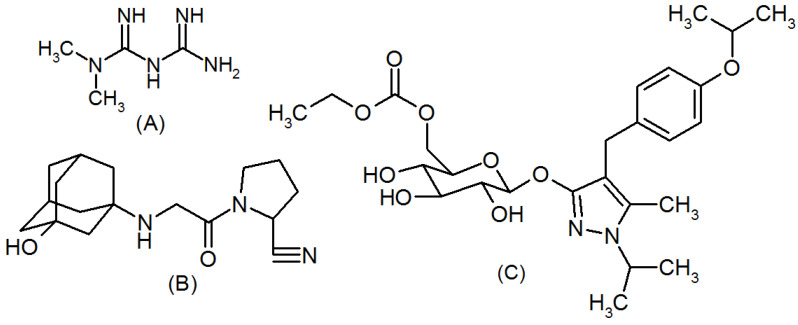
Chemical structures of metformin (**A**), vildagliptin (**B**), and remogliflozin (**C**).

Diabetes mellitus type 2 (DMT-2) is a result of pathophysiological modifications such as increased glucagon secretion in the liver and increased peripheral resistance to insulin, eventually leading to an increased blood glucose level [1,2]. Maintaining a normal blood glucose level is essential to avoid damage to the eyes, heart, kidney, and nervous system [3]. Metformin is a first-line oral hypoglycemic medicine for the management of DMT-2; however, with time, the use of an additional oral antidiabetic drug along with metformin is necessary for maintaining a normal HbA1C level [4,5]. Hence, a triple combination of metformin, vildagliptin, and remogliflozin with high glycemic control has been developed. Metformin (MF, Figure 1A) is highly beneficial in controlling the blood glucose level and reducing diabetic-associated complications. Physiologically, metformin reduces the storage of glucose in the liver by inhibiting hepatic gluconeogenesis. Metformin also reduces intestinal glucose uptake by enhancing the anabolism of glucose in the enterocytes of the gut in the absence of oxygen. It also increases the absorption and utilization of glucose in the peripheral tissue [6,7]. Various analytical procedures have been presented in scientific reports for the analysis of metformin using different types of samples, including spectrophotometry [8,9,10], HPLC [11,12], and LC–MS/MS [13]. Vildagliptin (VD, Figure 1B) acts by increasing the unbroken GLP-1 concentration by binding with dipeptidyl peptidase-4 (DPP-4) enzyme, which is accountable for the metabolism of GLP-1, thereby inhibiting hepatic gluconeogenesis by inhibiting glucagon secretion and increasing the secretion of insulin after the ingestion of food. Vildagliptin has shown good glycemic control with a reduced threat of hypoglycemic effect and increase in body weight, along with a protective effect on the heart [14,15,16,17,18]. Many assay procedures have been announced for the analysis of VD, such as spectrophotometric [19,20,21], spectrofluorometric [21], HPTLC [22], capillary electrophoresis [23], LC–MS/MS [24], and electrochemical [25] methods.

The function of the sodium–glucose cotransporter 2 (SGLT-2) found in the kidney is to reabsorb the glucose from the glomerular filtrate into the blood. Remogliflozin etabonate (RE, Figure 1C) inhibits the SGLT-2 enzyme and reduces the blood glucose level. It acts without involving insulin; hence, there is a low risk of insulin sensitivity. In addition, it reduces body weight by increasing the excretion of glucose through urine and other paths. In addition, RE is cardioprotective and nephron-protective; thus, it can be used in heart and kidney patients [26,27,28,29,30,31]. Various techniques are applied for RE determination, such as LC–MS/MS [31], spectrophotometric [32], HPTLC [32], and HPLC [33,34] methods. A binary formulation of ME and VD was analyzed using derivative spectrophotometric [35], HPLC-DAD [36,37], and capillary electrophoresis [38] methods. Other binary combinations containing ME and RE were concomitantly assayed by ratio derivative spectroscopic [39,40], HPLC-DAD [40], and UPLC [41] methods. Analysis of a VD and RE combination by spectrophotometric [42], HPLC [43,44], and LC–MS/MS [45] methods has been reported in the literature.

However, while reviewing scientific databases, no comprehensive analytical procedures were traced for the concomitant estimation of a ternary combination of MF, VD, and RE. Consequently, we established in this study, for the first time, a hydrophilic interaction liquid chromatography (HILIC) technique for the concurrent quantification of MF, VD, and RE ternary combinations within a single run in a short analysis time.

The utilization of experimental design in the establishment of analytical procedures is increasing due to the utilization of multivariate optimization of analysis conditions [46]. The utilization of an experimental design in developing robust analytical methods reduces the number of runs, time, and resources when compared to traditional univariate optimization. This is because an experimental design is based on predefined objectives and the identification of variable factors having a significant interactive effect on the analysis. The importance of experimental design is increased due to the incorporation of quality by design as a regulatory requirement of analytical method development used for the quality control of pharmaceutical formulations targeting the safety of patients, as part of pharmaceutical product development [47,48,49]. The literature features the use of surface response methodologies, such as central composite design for the development of the HILIC method [50,51]. Furthermore, Musaa et al. [52] reported the optimization of chromatographic conditions, by examining the effect of pH of the mobile phase, and percentage acetonitrile on the resolution between metformin, linagliptin, empagliflozin, and canagliflozin peaks as a response using face-centered central composite design (CCD). A chromatographic method was developed for the quantification of an antidiabetic formulation containing metformin using face-centered CCD, by optimizing the buffer pH, percentage acetonitrile, and percentage surfactant using the resolution as a response [53]. CCD, a response surface methodology (RSM), is a process optimization tool, which provides the direct and combined effects of variable parameters. Furthermore, face-centered CCD is better than circumscribed CCD and inscribed CCD because it reduces prediction errors and improves the estimation of effects [54]. Hence, in the present work, a face-centered central composite design with fewer runs was adopted to optimize the liquid chromatographic method for rapid simultaneous analysis of all three analytes. Moreover, the robustness of the method was verified using a two-level full factorial design.

## 2. Results and Discussion

### 2.1. Optimization of Chromatographic Conditions

The importance of the quality by design approach is to chromatographically separate the analytes using fewer runs with good resolution and peak shape. Hence, in the present work, a face-centered central composite design and a surface response design were exploited for the optimization of experimental conditions. In our previous research work on the establishment of chromatographic methods for the quantification of MF and VD, we observed a lower retention time for both analytes along with low resolution by RP-HPLC due to the hydrophilic nature of the analytes [37]. Hence, in the present work, an HILIC column was employed for the separation of compounds, as it retains the hydrophilic compounds due to the water layer present in the stationary phase and high percentage of organic solvent in the mobile phase [55]. A 150 mm HILIC column was chosen to build a rapid liquid chromatographic technique. The use of a large amount of phosphate salt results in precipitation in the column due to the high proportion of the organic phase in the mobile phase; thus, 20 mM phosphate buffer was utilized throughout the experiment. Vildagliptin had strong UV absorption only at 210 nm, while MF and RE also had good absorption; hence, 210 nm was chosen as the wavelength for further investigation. A preliminary study showed that using more than 70% acetonitrile in the mobile phase eluted MF after 7 min at a pH less than 5.5, whereas baseline separation between VD and MF was not achieved. Separation quality is important; thus, the typical key response is good resolution between critical analyte peaks [56,57,58]. Similar work has been reported on different drugs in which the resolution was the only response, while buffer pH, percentage acetonitrile, and flow rate had a significant effect on the response [51,52,53,58]. The three-level face-centered central composite design suggested by the software and the resolution results are tabulated below (Table 1).

Table 2 represents the quadratic equation coefficients along with their respective *p*-values. The coefficient values of all studied parameters showed an influence on the separation between the peaks. The first-order influence of all three parameters showed a substantial effect on the separation between VD and MF (*p* < 0.05), whereas the effect of all three parameters on the resolution between RE and VD was not significant, as confirmed by the *p*-values < 0.001 and >0.05, respectively. For the prediction of a response, a significant model is required [57]. A significant model with *p* < 0.05 indicates that one or more variable parameters have a significant influence on the mean response [59]. Furthermore, the amount of acetonitrile showed a negative effect, whereas the pH of the mobile phase and the flow rate showed a positive influence on the resolutions RS1 and RS2. The positive values indicated that the resolution was directly proportional, while the negative value indicated that the resolution was inversely related [60]. The interactive effect of the studied variables exhibited a substantial influence on the resolutions between RE and VD, as well as between VD and MF. The quadratic effect of acetonitrile concentration, pH, and flow rate had a substantial impact on the resolution between RE and VD, whereas only acetonitrile concentration and pH exhibited a substantial effect on the resolution between VD and MF. The reported methods for the optimization of chromatographic conditions for the separation of metformin with other drugs showed a much lower effect due to the use of RP-HPLC, where metformin was eluted first [52,53]. The assessed model *F*-values were found to be 26.06 and 33.40 (*p* < 0.0001) for RS1 and RS2, respectively. The *p*-values less than 0.05 indicate that the model was significant at the 95% confidence level [61]. In addition, the *F*-values for lack of fit were found to be 0.979 (*p* = 0.5427) and 3.77 (*p* = 0.1518) for RS1 and RS2, respectively, indicating that the model was adequate at the 95% confidence level [61]. Furthermore, the high *R*^2^ values, 0.967 and 0.974 for the resolutions of RE–VD and VD–MF, respectively, confirm the effective prediction of the resolution. Additionally, the adjusted *R*^2^ values of RS1 (0.923) and RS2 (0.945) were close to the predicted *R*^2^ values of 0.863 and 0.894, respectively. An accurate prediction by the model for the new measurements depends upon high predicted *R*^2^ values [61]. Adequate precision is represented by the signal-to-noise ratio; the values for RS1 (16.28) and RS2 (22.65) were higher than four [53,61], indicating that the suggested prototype is an adequate model and can be used to develop the design space to suggest the optimal conditions [61].

**Figure 2:**
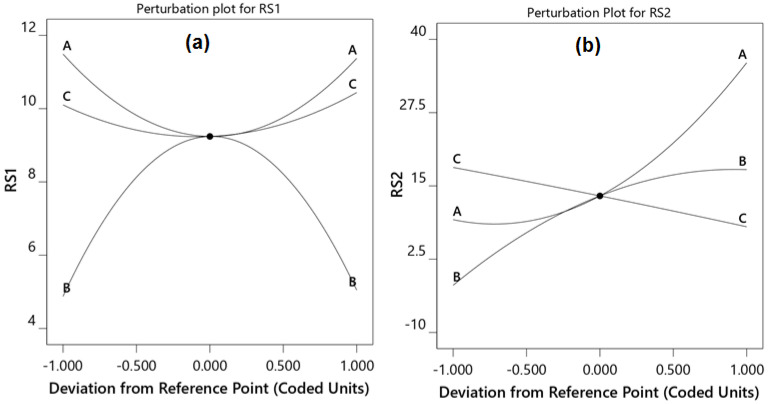
Perturbation plots showing the effect of the concentration of acetonitrile (A), pH of the mobile phase (B), and flow rate (C) on the resolutions of RE−VD (**a**) and VD−MF (**b**).

The perturbation plot for the resolution between RE and VD (Figure 2a) showed that an increase or reduction in the pH decreased the separation; however, the resolution was increased between RE and VD with an increase or decrease in the acetonitrile concentration and flow rate. Figure 2b shows that increases in the acetonitrile concentration of the mobile phase and pH improved the resolution between VD and MF, whereas the flow rate showed the opposite effect.

**Figure 3:**
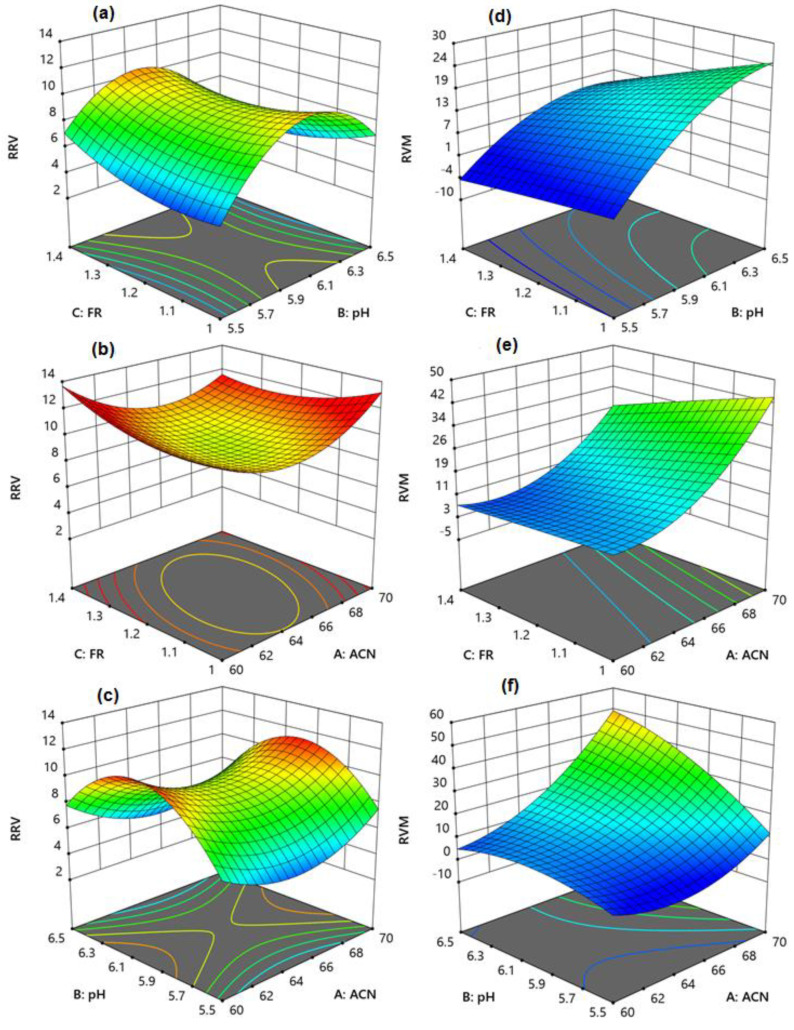
The 3D response surface models showing the effect of concentration of acetonitrile (A), pH of the mobile phase (B), and flow rate (C) on the resolutions of RE−VD (**a**–**c**) and VD−MF (**d**–**f**).

The 3D surface response model shows the combined effects of different studied parameters on the resolution of the peaks. The interactive effects of all three studied parameters showed a slight influence on the resolution between RE and VD, because of the less hydrophilic nature of RE and VD (Figure 3a–c). On the other hand, the interactive effect of all three parameters had a significant effect on the resolution between VD and MF. The resolution was improved with an increase in pH (Figure 3d) and acetonitrile concentration (Figure 3e), but with a decrease in the flow rate.

Similarly, increasing the mobile phase pH and acetonitrile concentration increased the separation between VD and MF (Figure 3f), which is in agreement with the separation of hydrophilic analytes in HILIC conditions [55]. As a result, an intermediate pH of 6, acetonitrile concentration of 65% *v*/*v*, and flow rate of 1.2 mL/min were identified as excellent for improved resolution; however, the analysis duration was greater than 4 min, with resolution values of close to 10 and 12 for RS1 and RS2, respectively.

**Figure 4:**
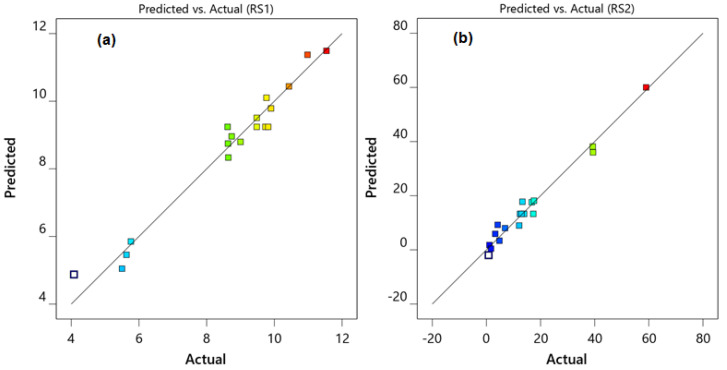
Diagnostic plots for resolutions of RE−VD (**a**) and VD−MF (**b**) showing the comparison of actuals, against predicted resolution values.

**Figure 5:**
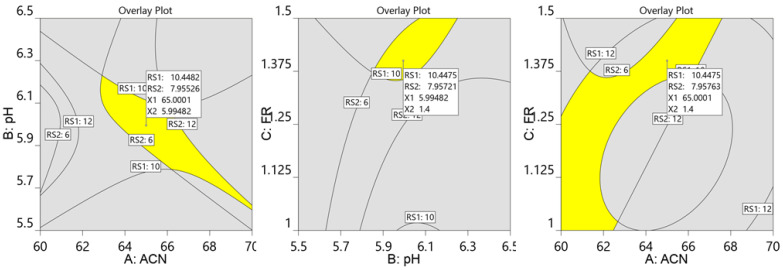
Overlay plot of chromatographic conditions suggested by the model. ACN: percentage acetonitrile; FR: flow rate.

Furthermore, the actual resolution values and the mathematically predicted resolution values were nearly identical and lay on the diagonal contour (Figure 4a,b). As a result, the quadratic polynomial equation generated could be deemed acceptable for predicting experimental conditions. The following experimental conditions with a desirability value of 0.998 and a short analysis time were selected by the software from among the 26 conditions: 64.9% *v*/*v* acetonitrile, mobile phase pH of 6.0, and flow rate of 1.4 mL/min (Figure 5).

**Figure 6:**
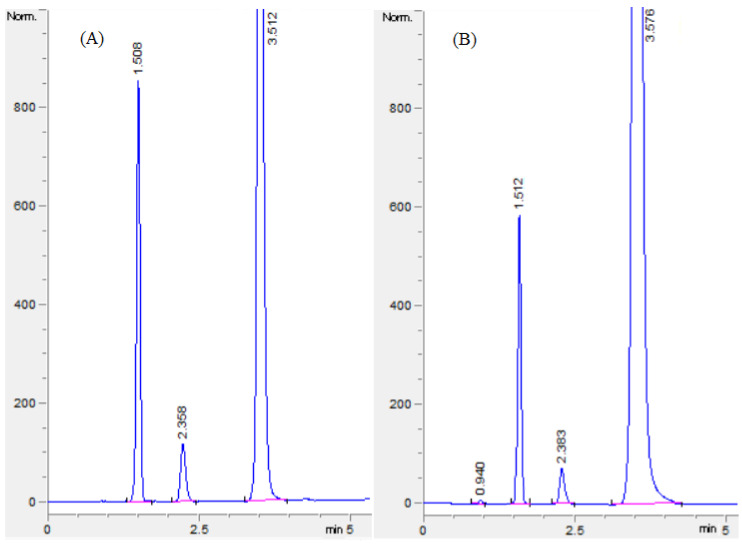
Representative chromatograms of standard solution (**A**) and formulation solution (**B**). Retention times: RE = 1.5 min, VD 2.3 = min, and MF = 3.5 min.

The suggested optimal chromatographic condition for the analysis of RE, VD, and MF was validated by performing five experiments using the HILIC column and the mobile phase comprising 65% acetonitrile and 35% 20 mM phosphate buffer (pH adjusted to 6 with orthophosphoric acid). The detection wavelength was set to 210 nm and the mobile phase flow rate was adjusted to 1.4 mL/min. The mean resolutions for RS1 and RS2 were calculated and found to be 7.78 and 10.35, respectively. The practically determined resolution values were in agreement with the predicted resolution values (7.95 and 10.44, respectively). Hence, using the optimized chromatographic conditions, RE, VD, and MF were effectively separated within 3.6 min with good peak shape (Figure 6A,B). Therefore, both standard solutions and formulation solutions were analyzed using the optimized conditions.

### 2.2. Validation of HILIC Procedure

#### 2.2.1. System Suitability Tests

The mean retention time, peak area, resolution, theoretical plate, and peak asymmetry, along with their standard deviations, were calculated, and the results are presented in Table 3. For all parameters, the percentage relative standard deviation was <2. Furthermore, the resolution and theoretical plate were more than 1.5 and 2000, respectively, with a peak asymmetry of roughly one.

#### 2.2.2. Linearity of Calibration Curve

RE, VD, and MF demonstrated linearity in a series of solutions consisting of 20–150 µg/mL, 10–75 µg/mL, and 50–750 µg/mL, respectively. The calibration curve was created using seven diverse concentrations in the range against the corresponding peak area of the analytes (Supplementary Figure S1). The regression equation of the standard curve was determined using Microsoft Excel software, while the intercept slope and regression coefficient were computed; the results are tabulated below.

#### 2.2.3. Sensitivity

The DL and QL were calculated using calibration curve parameters (Table 3). The low DL and QL confirmed that the different ratios of RE, VD, and MF present in the formulation can be estimated using the proposed HPLC method. 

#### 2.2.4. Precision and Accuracy

The precision of the analysis was stated as the percentage relative standard deviation (%RSD), and the mean %RSD was found to be 0.70–1.82%, 0.93–1.75%, and 0.91–1.54% for RE, VD, and MF respectively, certifying the precision of the developed HILIC procedure. 

The mean percentage recovery ranged from 98.61–100.65%, 98.45–100.20%, and 98.14–101.08% for RE, VD, and MF, respectively. The percentage relative errors were also within the tolerable range, confirming the accuracy of the HILIC technique. (Table 4)

#### 2.2.5. Selectivity

The excipient chromatogram did not show any disturbing peaks at the retention time of all the three analytes. Furthermore, the chromatograms of formulation solutions of RE, VD, and MF had well-separated neat discrete peaks, demonstrating the selectivity of the proposed HILIC technique (Figure 6B).

#### 2.2.6. Stability of Solutions

The RE was stable for more than seven days in the refrigerator, whereas a significant change was observed in the area of VD and MF peaks after the fifth day. The peak area of working standard solutions kept in the autosampler was unchanged for at least 8 h, which is sufficient to complete daily analysis.

#### 2.2.7. Robustness

**Figure 7:**
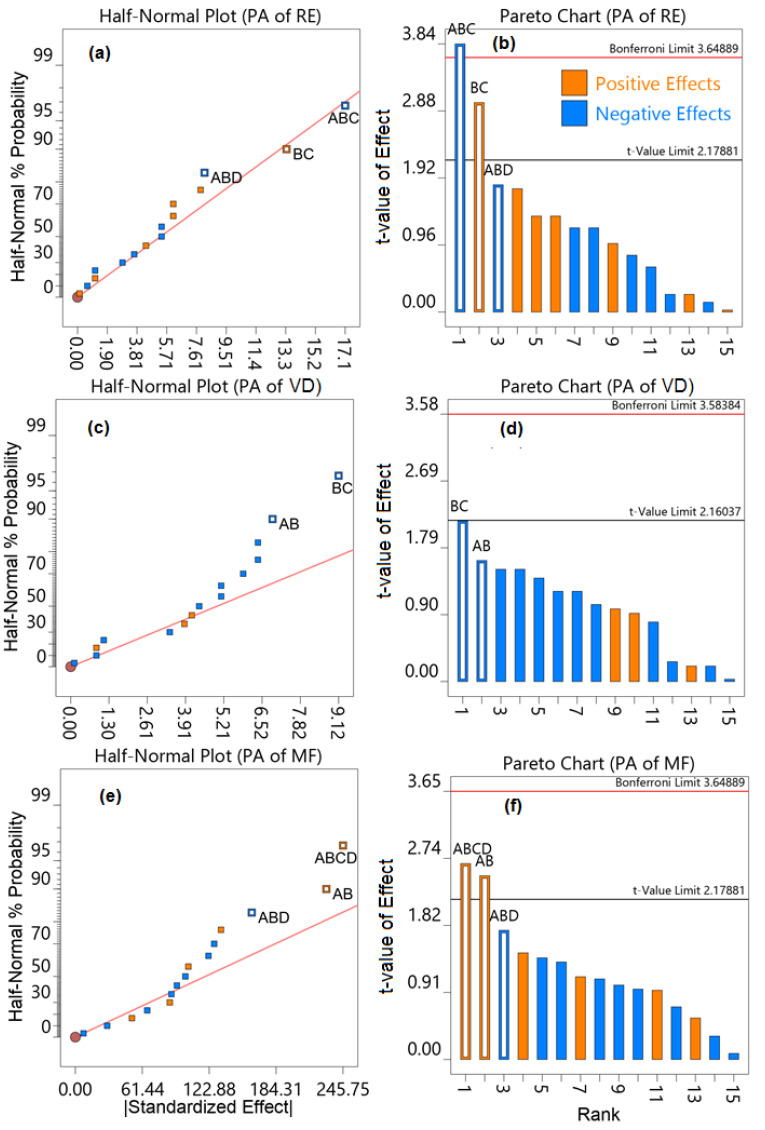
Half-normal probability plots and Pareto charts showing the effect of the concentration of acetonitrile (A), pH of the mobile phase (B), flow rate (C), and wavelength of the detector (D) on the peak area of RE (**a**,**b**), VD (**c**,**d**), and MF (**e**,**f**). PA: peak area.

Full factorial design (FFD) was used for the determination of robustness of the method as the number of factors was four. The number of levels is two in chromatography [61,62,63]. Pareto charts and half-normal probability plots (Figure 7) generated from the 16 experiments demonstrate the effects of independent and collective effects of variable parameters on the assay results (Supplementary Materials Table S1). The parameter values beyond the Bonferroni perimeter were extremely significant; however, the values beyond the *t*-value were significant [53,64]. In the developed HILIC method, a slight variation in the single studied parameters did not show a significant effect on the peak area of all three analytes, as none of the single parameter effects were above the *t*-value limit. The combined effect of concentration of acetonitrile, pH, and wavelength had a considerable influence on the peak area of RE, whereas the combined effect of all four parameters had a large influence on the peak area of MF. However, in the robustness testing, the influence of factor interactions could be neglected [51], indicating the robustness of the developed HILIC method.

### 2.3. Application of the HPLC Method for the Analysis of Formulation

Two different concentrations of the formulation were analyzed in triplicate, and the outcomes are presented in Table 5. The amount determined by the developed HILIC method was in agreement with the amount mentioned on the formulation label. Furthermore, the chromatogram of the formulation (Figure 6B) did not show any interfering peaks of tablet excipients. Selectivity and accuracy were confirmed by quantifying the added amount to the above-analyzed samples using the standard addition technique. The mean percentage recovery and relative error were well within the acceptable range, (Table 5) an indication of the selectivity and accuracy of the established HILIC method for the concomitant estimation of RE, VD, and MF in the formulation.

## 3. Materials and Methods

### 3.1. Materials

The standard API of metformin HCl (99.7%) and vildagliptin (99.9%) was supplied by Biokemics India Limited (Hyderabad, India). An analytically pure standard of remogliflozin etabonate (99.8%) was purchased from Metrochem (Hyderabad, India). The mobile phase was made with analytical-grade potassium dihydrogen phosphate and dipotassium hydrogen phosphate obtained from Sigma Aldrich Chemie (Saint Louis, MO, USA). Analytically pure HPLC-grade acetonitrile was procured from Fischer Scientific (Fisher Scientific (Loughborough, UK). Orthophosphoric acid (85% *v*/*v*) used for the adjustment of pH was purchased from S.D. Fine Chem Ltd. (Mumbai, India). Two formulations containing 100 mg of RE and 50 mg of VD with 500 and 1000 mg of MF were purchased from the Indian market.

### 3.2. Instrumentation and Software

Analysis was carried out using a computer system coupled to a high-performance liquid chromatography system (Agilent Technologies, Waldbronn, Germany) with an autosampler, vacuum degasser, quaternary pump, and photodiode array detector. The column used was the Acclaim mixed-mode HILIC-1 column (150 mm × 4.6 mm, i.d. 5 µm, 120 A°) (Thermo Fisher Scientific company, Göteborg, Sweden). Chem station software (Ver B.04.03.SP1 Agilent Technologies, Santa Clara, CA, USA) was used to manage the analyte elutes. The pH of the mobile phase was adjusted using a pocket pH meter (Martini Instruments, Gallarate, Italy). Design Expert 12 (Stat-Ease, Minneapolis, MN, USA) was used to create the central composite design.

### 3.3. Preparation of Primary Stock and Working Standard Solutions

Primary stock solutions of MF, VD, and RE were arranged separately by adding 50 mL of methanol to 50 mg of MF, VD, or RE to attain a concentration of 1 mg/mL. A sufficient volume of the mobile phase was transferred to the primary standard solutions to reduce the concentrations of the analytes to the working standard solutions before injecting them into the HPLC system.

### 3.4. Preparation of Sample Solution

Twenty tablets comprising MF (500 mg), VD (50 mg), RE (100 mg), MF (1000 mg), VD (50 mg), and RE (100 mg) were weighed and powdered. Powder corresponding to MF (50 mg), VD (5 mg), RE (10 mg), MF (100 mg), VD (5 mg), and RE (10 mg) was weighed separately and shifted into a 50 mL graduated flask containing methanol (25 mL). The mixture was subjected to sonication for 10 min, to entirely dissolve the drugs in the solvent. Then, the solution was filtered into another graduated flask, and the total volume was adjusted to 50 mL with the same solvent. The required quantity of the mobile phase was transferred to an aliquot of the sample solution to adjust the concentration of the compounds to the linearity range just before estimation by the optimized HILIC procedure.

### 3.5. Chromatographic Procedure

The separation of all analytes (MF, VD, and RE) was executed using an HILIC column. Phosphate buffer (20 mM) was prepared by adding 86.6 mL of 1 M potassium dihydrogen phosphate solution to 13.2 mL of 1 M dipotassium hydrogen phosphate, and the total volume was adjusted to 1 L using water, while the final pH of the solution was adjusted to 6 using orthophosphoric acid. The optimized mobile phase comprising acetonitrile (65% *v*/*v*) and 20 mM phosphate buffer (35% *v*/*v*, pH 6) was forced isocratically with a flow rate of 1.4 mL/min. The detector wavelength was set to 210 nm to monitor the elute. The analysis was performed by injecting 20 µL of compound solutions at room temperature.

### 3.6. Optimization by Central Composite Design

The chromatographic conditions were improved using a central composite design for the baseline separation of RE, VD, and MF peaks with excellent peak shape in less time. The three critical factors selected were acetonitrile concentration (60, 65, and 70%), pH of the mobile phase (5.5, 6, and 6.5), and flow rate (1, 1.2, and 1.4 mL/min) at three levels (−1, 0, +1). The wavelength was set to 210 nm, and the concentration of buffer used was 20 mM throughout the experiment. The resolutions of RE–VD (RS1) and VD–MF (RS2) were considered as the response for the optimization. The 18 chromatographic experiments comprising four center position values were executed in random order as per the central composite design proposed by the Design-Expert software (Table 1). Furthermore, because the HILIC column took longer to equilibrate with the mobile phase, the HILIC column was washed with the mobile phase for 30 min during the change in mobile phase composition to achieve an equilibrium between the HILIC stationary phase and the mobile phase. Specifically, 200 µg/mL MF, 50 µg/mL VD, and 100 µg/mL RE were used in the tests. Statistical analysis was used to determine the optimal parameters using the ANOVA, and the following polynomial equation was generated using the quadratic model:

$$\mathrm{RS}=\theta_{0}+\theta_{1}X_{1}+\theta_{2}X_{2}+\theta_{3}X_{3}+\theta_{4}X_{1}^{2}+\theta_{5}X_{2}^{2}+\theta_{6}X_{3}^{2}+\theta_{7}X_{1}X_{2}+\theta_{8}X_{1}X_{3}+\theta_{9}X_{2}X_{3},$$

where RS is a response (resolution between two adjacent peaks), θ_0_ is a constant, and θ_1_–θ_9_ are coefficient values of the three parameters (amount of acetonitrile (X_1_), mobile phase pH (X_2_), and mobile phase flow rate (X_3_)). The square values of X_1_ to X_3_ are quadratic terms of each parameter, while X_1_X_2_, X_1_X_3_, and X_2_X_3_ show the combined effects of variable factors.

To achieve high chromatographic performance conditions, a numerical optimization design space with a desirability of 1 was constructed.

### 3.7. Validation of the Chromatographic Method

The developed HILIC procedure was validated for various factors such as linearity, sensitivity, accuracy, precision, selectivity, stability, and robustness. The validation was performed by analyzing the mixture of all three analytes in triplicate, as per the validation protocol provided by the International Council for Hominization (ICH) [65].

Different system suitability parameters were calculated by investigating the standard solutions of MF, VD, and RE with concentrations of 200 µg/mL, 50 µg/mL, and 100 µg/mL, respectively. All system suitability factors (resolution, peak area, theoretical plates, and peak tailing) were evaluated by computing the percentage relative standard deviation of six determinations. According to the ICH guidelines, the acceptable range for the resolution is above 1.5, that for theoretical plates is above 2000, that for peak tailing is 0.9–1.2, and that for peak area is below 2 [65].

Six working standard solutions of MF, VD, and RE with concentration ranges of 50–750 µg/mL, 10–75 µg/mL, and 20–150 µg/mL, respectively, were arranged by conveying the essential quantity of stock solution. The compound solutions were chromatographed by the standardized HILIC method. The average peak areas of all analytes were documented from the three determinations, and a linearity curve was generated against the respective concentrations of MF, VD, and RE. 

The detection limit (DL) and quantification limit (QL) were computed to evaluate the sensitivity of the method calibration curve factors. DL and QL of the proposed method were calculated using calibration parameters. DL and QL were determined by constructing the calibration curves in triplicate, and the residual standard deviation and mean slope were calculated. DL and QL were 3.3 fold and 10 fold the residual standard deviation of the calibration curve to the average slope of the linearity curve, respectively.

The precision of the HILIC process was determined considering the repeatability (within-day precision) and intermediate precision (between day precision). Three concentrations of all three analytes (low, medium, and high) covering the full linearity range were quantified thrice on the same day for the within-day precision and on three consecutive days for the between-day precision. The concentrations used were 20, 75, and 150 µg/mL for RE; 10, 40, and 75 µg/mL for VD; and 50, 350, and 750 µg/mL for MF. The precision was communicated as the percentage relative standard deviation of the calculated amount. The accuracy of the HILIC method was evaluated by calculating the percentage assay and percentage relative error for both within-day and between-day accuracy. 

Selectivity of the HILIC method was determined by matching the chromatogram of standard solutions of analytes with the chromatogram of the tablet excipients. The required amounts of tablet excipients, microcrystalline cellulose, magnesium stearate, lactose, starch, and silica were dissolved in methanol and filtered. The standard solution and blank solution were analyzed using optimized HILIC conditions. Furthermore, the selectivity and accuracy were confirmed by the standard addition method. A weighed quantity of standard solutions was transferred to the previously quantified formulation solution, and the percentage recovery was computed. 

To study the stability of standard solutions of RE, VD, and MF, 1 mg/mL of the solutions in methanol stored in the refrigerator at 4 °C was mixed using the mobile phase to bring the concentration of drugs within the calibration curve range and were analyzed every day. For benchtop stability, working standard solutions prepared in the mobile were evaluated by analyzing the samples over 8 h on the same day. To assess the stability of analyte solutions, the peak area was compared.

The robustness of the HILIC technique was determined by performing assays using standard solutions of MF, VD, and RE while slightly changing the optimized chromatographic conditions. A multivariate approach, the full factorial n^k^ factorial design, where n is the number of levels and k is the number of variable factors, was adopted to determine the impact of variation in the chromatographic conditions on the peak area of the analytes [61,62]. The 16 (2^4^) experiments at two levels with four variable parameters were conducted as per the order suggested by the software. Slight changes in the wavelength (±2 nm), flow rate (±0.1 mL/min), pH (±0.1), and mobile phase composition (±2 mL of acetonitrile) were applied, and the standard analyte solutions were analyzed.

## 4. Conclusions

The hydrophilic interaction liquid chromatography technique was employed to establish a rapid and reproducible analytical procedure for the concurrent determination of remogliflozin, vildagliptin, and metformin in formulations. A face-centered central composite design applied for the optimization of chromatographic conditions concluded that the influence of acetonitrile concentration was negative, whereas buffer pH and flow rate had a positive on the resolution between analyte peaks. The overlapping plot with a desirability value of 0.998 allowed the identification of optimal chromatographic conditions, which separated all three analytes within 4 min. It is worth saying that the starting conditions turned out to be the best. The robustness study confirmed that the slight variation of the studied independent variables did not affect the peak area of the analytes. The high percentage recovery of the added analyte and percentage assay of the formulation confirmed the selectivity and accuracy of the developed HILIC method. As a result, the established rapid, accurate, precise, and robust HILIC approach for concurrent determination of RE, VD, and MF in pharmaceutical formulations can be used for frequent quality control examinations.

## Figures and Tables

**Table 1:** Seventeen experiments suggested by face-centered CCD for the optimization of chromatographic conditions.

Pattern-Coded Value	Run	Factor 1: Percentage Acetonitrile	Factor 2: pH	Factor 3: Flow Rate (mL·Min^−1^)	Resolution between RE and VD	Resolution between VD and MF
+ + +	1	70	6.5	1.4	5.76	39.29
− − +	2	60	5.5	1.4	9.90	4.81
0 0 0	3	65	6	1.2	9.74	13.32
0 0 0	4	65	6	1.2	9.62	12.98
0 0 0	5	65	6	1.2	9.48	13.93
+ − −	6	70	5.5	1	8.64	16.77
0 + 0	7	65	6.5	1.2	5.50	13.30
0 − 0	8	65	5.5	1.2	4.08	0.81
− − −	9	60	5.5	1	5.63	1.11
0 0 0	10	65	6	1.2	9.82	12.41
0 0 −	11	65	6	1	9.77	17.57
− + +	12	60	6.5	1.4	8.74	1.69
− + −	13	60	6.5	1	8.63	12.09
+ 0 0	14	70	6	1.2	10.98	39.38
− 0 0	15	60	6	1.2	11.54	4.15
+ + −	16	70	6.5	1	9.48	59.01
+ − +	17	70	5.5	1.4	9.00	3.26
0 0 +	18	65	6	1.4	10.43	7.89

**Table 2:** Polynomial equation coefficients with corresponding *p*-values for peak resolution.

Coefficient Terms	Resolution between RE and VD (RS_1_)		Resolution between VD and MF (RS_2_)	
	Coefficient Value	*p*-Value	Coefficient Value	*p*-Value
Constant	+9.24	<0.0001	+1427.372	<0.0001
ACN ^a^	−0.058	0.7451	−61.984	<0.0001
pH	+0.086	0.6309	+88.987	<0.0001
Flow rate	+0.168	0.3571	+337.112	0.0026
ACN × pH	−0.529	0.0252	+3.520	0.0001
ACN × flow rate	−0.967	0.0010	−3.317	0.0327
pH × flow rate	−1.03	0.0007	−25.406	0.0837
ACN^2^	+2.19	0.0002	+0.365	0.0033
pH^2^	−4.28	<0.0001	−22.299	0.0357
Flow rate^2^	+1.03	0.0145	−2.570	0.9640

^a^ Amount of acetonitrile in the mobile phase.

**Table 3:** System suitability and regression analysis results.

Parameters	RE	VD	MF
System suitability results			
Retention time ± SD	1.51 ± 0.021	2.35 ± 0.029	3.54 ± 0.065
Peak area ± SD	5765.5 ± 46.84 ^a^	824.29 ± 7.34 ^b^	9678.47 ± 85.75 ^c^
Resolution ± SD	-	7.85 ± 0.05 ^d^	10.40 ± 0.08 ^e^
Tailing factor ± SD	1.12 ± 0.022	1.05 ± 0.013	1.15 ± 0.023
Theoretical plate ± SD	7544.16 ± 65.24	10,908.64 ± 78.98	20,506.24 ± 96.47
Linearity			
Linearity range (µg/mL)	20–150	10–75	50–750
Slope	57.915	17.171	50.487
Intercept	48.462	−46.048	−237.84
Regression coefficient (*r*^2^)	0.9992	0.9988	0.9997
Sensitivity			
DL (µg/mL)	4.73	2.81	1.48
QL (µg/mL)	14.34	8.54	43.88

SD: standard deviation; DL: detection limit; QL: quantification limit; ^a^ 100 µg/mL; ^b^ 50 µg/mL; ^c^ 200 µg/mL; ^d^ resolution between RE and VD; ^e^ resolution between VD and MF.

**Table 4:** Precision and accuracy results of the developed HILIC method.

Drug	Within-Day					Between-Day			
	Amount (µg/mL)	Amount Found Mean (*n* = 3) ± SD	% RSD	% Recovery	% RE	Amount Found Mean (*n* = 9) ± SD	% RSD	% Recovery	% RE
RE	20	19.86 ± 0.14	0.70	99.30	−0.70	20.13 ± 0.23	1.14	100.65	0.65
	75	74.18 ± 1.05	1.42	98.91	−1.09	74.25 ± 1.35	1.82	99.00	−1.00
	150	148.64 ± 2.33	1.57	99.09	−0.91	147.92 ± 2.09	1.41	98.61	−1.39
VD	10	10.02 ± 0.12	1.20	100.20	0.20	9.86 ± 0.16	1.62	98.60	−1.40
	40	39.65 ± 0.37	0.93	99.13	−0.88	39.38 ± 0.69	1.75	98.45	−1.55
	75	74.24 ± 0.82	1.10	98.99	−1.01	74.46 ± 1.13	1.52	99.28	−0.72
MF	50	49.16 ± 0.51	1.04	98.32	−1.68	49.07 ± 0.52	1.06	98.14	−1.86
	350	346.73 ± 5.34	1.54	99.07	−0.93	353.79 ± 4.66	1.32	101.08	1.08
	750	745.04 ± 6.79	0.91	99.34	−0.66	746.21 ± 7.08	0.95	99.49	−0.51

SD: standard deviation; %RSD: percentage relative standard deviation; %RE: percentage relative error.

**Table 5:** Assay results of formulations and percentage recovery by standard addition method.

Label Claim (mg/Tablet)	Amount Taken (µg·mL^−1^)	Amount Found (µg·mL^−1^)	% Purity	%RSD
Formulation 1 (VD 50 mg + RE 100 mg + MF 500 mg)	VD 20 RE 40 MF 200	20.12 39.73 197.58	100.60 99.33 98.79	1.15 1.48 1.56
Formulation 2 (VD 50 mg + RE 100 mg + MF 1000 mg)	VG 50 RE 100 MF 500	49.62 99.17 495.37	99.24 99.17 99.07	0.93 1.72 1.08
Standard addition method			Recovery %	
Amount of VD added (µg·mL^−1^) to formulation solution 1	10	10.01	100.10	0.83
	20	19.84	99.20	1.44
	30	29.47	98.23	1.31
Amount of RE added (µg·mL^−1^) to formulation solution 1	20	19.95	99.75	1.28
	40	39.28	98.20	1.72
	60	59.04	98.40	0.76
Amount of MF added (µg·mL^−1^) to formulation solution 1	100	98.83	98.83	1.34
	200	198.11	99.06	1.77
	400	396.45	99.11	0.68

## Data Availability

The data generated during this work are included in the manuscript and its Supplementary Materials.

## Supplementary Materials

The following supporting information can be downloaded at: https://www.mdpi.com/article/10.3390/molecules27103135/s1, Figure S1: Calibration curves for standard solutions of remogliflozin, vildagliptin, and metformin; Table S1: Two-level full factorial design for robustness study and results.

## Sample Availability

Samples of the compounds are not available from the authors.
