# Supplementary material for: An Experimental Design Approach to Quantitative Expression for Quality Control of a Multicomponent Antidiabetic Formulation by the HILIC Method
Source: Molecules. 2022 May 13;27(10):3135. doi: 10.3390/molecules27103135 (PMC9148089; doi:10.3390/molecules27103135)
Supplement: Supplementary file 1 [file molecules-27-03135-s001.zip › molecules-1702014-supplementary.pdf]

Supplementary Materials

An Experimental Design Approach to Quantitative Expression for Quality Control of a Multicomponent Antidiabetic Formulation by the HILIC Method

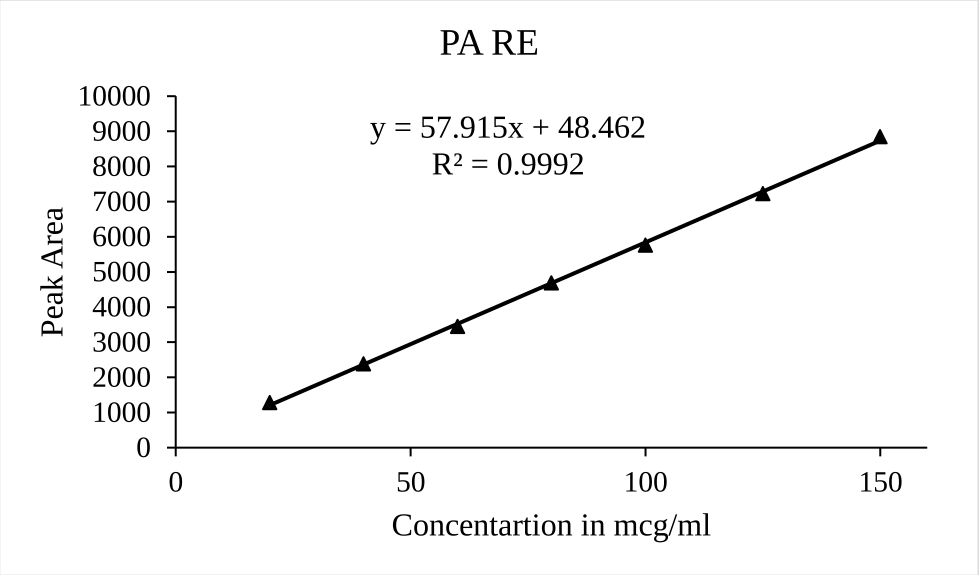

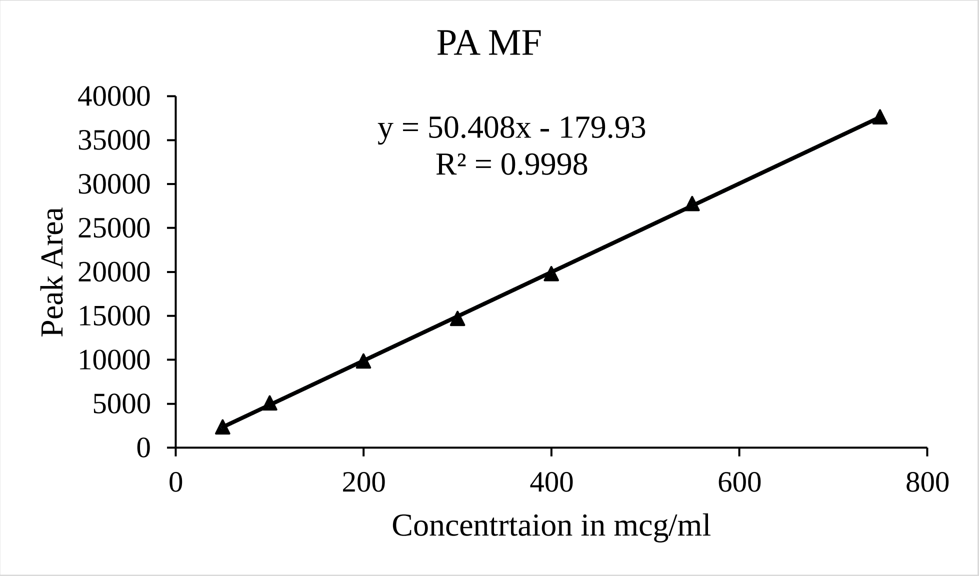

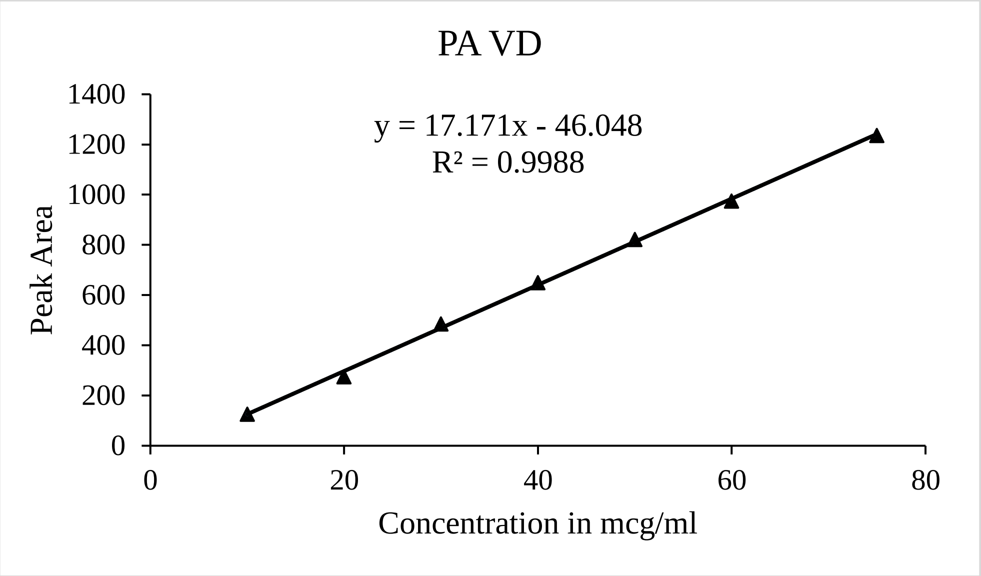

Figure S1. Calibration curves for remogliflozin (RE), vildagliptin (VD and metformin (MF).

Table S1. Two-level full factorial design for robustness study and results.

	Factor 1	Factor 2	Factor 3	Factor 4	Response 1	Response 2	Response 3
**Run**	**A: Amount of acetonitrile**	**B: Mobile phase pH**	**C:Flow Rate**	**D:Wave Length**	**Peak Area of Remogliflozin**	**Peak Area of Vildagliptin**	**Peak Area of Metformin**
1	67	5.8	1.5	212	5785	834	12439
2	67	6.2	1.3	208	5770	833	12900
3	63	5.8	1.5	208	5760	819	12790
4	63	6.2	1.5	208	5780	820	12537
5	67	6.2	1.5	208	5786	827	12509
6	67	5.8	1.5	208	5761	815	12645
7	67	5.8	1.3	212	5755	816	12666
8	63	5.8	1.3	208	5785	818	12555
9	67	6.2	1.3	212	5764	824	12309
10	63	5.8	1.3	212	5789	813	12679
11	63	5.8	1.5	212	5746	822	12780
12	67	6.2	1.5	212	5765	800	12546
13	63	6.2	1.3	212	5757	834	12500
14	63	6.2	1.3	208	5744	839	11900
15	67	5.8	1.3	208	5759	830	12102
16	63	6.2	1.5	212	5775	823	12435
